# Self-esteem in stabilized individuals with chronic schizophrenia: association with residual symptoms and cognitive functioning

**DOI:** 10.1007/s00406-022-01538-x

**Published:** 2023-01-05

**Authors:** Alex Hofer, Falko Biedermann, Alexandra Kaufmann, Georg Kemmler, Nicole M. Pfaffenberger, Nursen Yalcin-Siedentopf

**Affiliations:** Division of Psychiatry I, Department of Psychiatry, Psychotherapy, Psychosomatics and Medical Psychology, Medical University Innsbruck, Anichstr. 35, 6020 Innsbruck, Austria

**Keywords:** Schizophrenia, Self-esteem, Psychopathology, Cognition

## Abstract

Low self-esteem is regarded as a barrier to recovery from schizophrenia and the identification of factors affecting this psychological characteristic may help to implement effective therapeutic interventions. To this end, the present study aimed to assess whether residual symptoms of the disorder and performance on a comprehensive neuropsychological test battery might differently impact self-esteem among 70 stabilized outpatients with chronic schizophrenia from public outpatient mental health services. Self-esteem inter-correlated with the severity of overall symptomatology, affective and negative symptoms, with premorbid intelligence, and with performance in the domains of verbal learning and memory, visual memory, working memory, and verbal fluency. Residual affective symptoms, premorbid intelligence, and female sex predicted poorer self-esteem in multiple linear regression analysis. The findings of this study implicate that next to psychological interventions therapeutic strategies that specifically target affective symptoms of schizophrenia may have a beneficial impact on patients’ self-esteem.

## Introduction

While the main focus in the treatment of schizophrenia has for a long time been put on a reduction in the severity of positive symptoms followed by an improvement of the negative and cognitive symptoms of the disorder (clinical recovery), in the last decade, increased emphasis has been placed on patients’ functioning and quality of life. This concept of personal recovery builds on the five processes of connectedness, hope and optimism, identity, meaning and purpose, and empowerment (CHIME) [1] and applies irrespective of limitations caused by the illness. From this perspective, self-esteem, i.e. a person’s evaluation of or attitude toward him or herself [2], which is widely recognized as a central component of psychological health and well-being, plays a key role in recovery [3].

Although not uniformly low, several studies have documented that self-esteem is compromised among people with psychotic disorders, including those at clinical high-risk [4] as well as individuals experiencing a first episode of psychosis [5] or suffering from chronic schizophrenia [6]. Low self-esteem has, for example, been associated with poorer premorbid social adjustment [7, 8], the development [7] and persistence of delusions [9, 10], more severe negative symptoms [11], depression [8, 12], a higher risk of suicidal ideation [13], more relapses [14], and poorer quality of life [15, 16] and functional outcomes [17]. It should also be noted that low self-esteem resulting from feelings of inferiority has been suggested to serve as a link between anomalous self-experiences and depressive symptoms as well as suicidality in these patients [18–21]. In addition, previous studies reported on inter-correlations between low self-esteem and both poor medication [22, 23] and psychosocial treatment adherence [24] as well as high internalized stigma [6, 25]. Most studies point to lower self-esteem in female patients, whereas other sociodemographic variables are hardly related to self-esteem [7, 26–28].

Research on a possible link between self-esteem and neurocognitive dysfunctions in schizophrenia is generally limited and equivocal. For example, in an early study by Brekke et al. [29], patients with poor executive functioning displayed a large positive and statistically significant association between psychosocial functioning and self-esteem, while those with intact executive functioning did not. Wittorf et al. [30], in turn, could not replicate this finding but rather reported on a bivariate correlation between both attention and memory skills and self-esteem, and Wang et al. [31] found inhibition of attention to predict self-esteem.

The reasons for low self-esteem in people suffering from schizophrenia are complex. Next to the high prevalence of childhood adversity experienced by this group [32], which is generally known to have a significant negative impact on self-esteem [33], illness-related impairments in regards of both school and vocational education as well as the establishment of stable social relationships might also interfere negatively with self-esteem [34, 35]. Likewise, difficulties in adapting and in coping with stress and with the illness in general could contribute to low self-esteem and vice versa [36]. Finally, a possible negative effect of antipsychotic medication on patients’ self-esteem has to be taken into account. On the one hand, high dopamine D_2_ receptor occupancy has been found to negatively impact well-being [37] and thus self-esteem [15], while on the other hand, stigmatizing side effects such as extrapyramidal motor symptoms [8, 38] and weight gain [28] are also of major relevance in this context.

In general, self-esteem has been suggested to be both a state and a trait measure and it clearly depends on an individual’s subjective evaluation, whether and how strongly certain circumstances, e.g., episodes of psychotic relapse, affect state self-esteem [39]. In view of these complex relationships, we investigated a sample of chronically ill individuals with schizophrenia spectrum disorders living within the context of community-based environments who had been stable both from a symptomatic and a medication perspective for a minimum of 6 months before study inclusion. Our goals were to (1) characterize self-esteem in a relatively large sample of regular attendees of public outpatient mental health services, i.e. in a group of patients one is interested in when evaluating the long-term management of people with schizophrenia, and (2) assess the association of self-esteem with residual symptoms of the disorder and neurocognitive skills. We hypothesized that the majority of study participants would indicate a clinically significant problem with self-esteem and that lower self-esteem scores would correlate with a greater severity of symptoms and neurocognitive impairment.

## Materials and Methods

### Study population

We performed a cross-sectional study including 70 patients from public outpatient mental health services between the ages of 18 and 60 who met the diagnostic criteria of schizophrenia spectrum disorders according to ICD-10 (paranoid schizophrenia: *n* = 41, residual schizophrenia: *n* = 22, simplex schizophrenia: *n* = 2, schizoaffective disorder: *n* = 5). Diagnoses were confirmed using chart information and reports from clinicians who had treated these patients. Study participants did not suffer from any other axis I disorder, severe somatic illnesses including a history of moderate or severe brain injury, or low intellectual ability. At the time of investigation, they were on fixed medication regimens since at least 6 months and there had not been any essential change in psychopathology during this period as judged by the treating psychiatrists. Only native German speakers were included into the study.

A trained schizophrenia research team registered sociodemographic and clinical data and performed ratings including the Global Assessment of Functioning Scale (GAF) [40]. Raters were blind to the results of neurocognitive testing. Cognitive assessments were done by a psychology PhD who was blind to scores on the other measures.

### Self-esteem

The Index of Self-Esteem (ISE) [41] is a 25-item scale to measure the degree, severity, or magnitude of a problem one has with self-esteem. Twelve items are reverse-scored. Examples include “I feel that I need more self-confidence” (item 8), “I feel that I bore people” (item 13), or “I think that if I could be more like other people I would have it made” (item 17). Each item is rated on a scale from 1 to 7 (1 = none of the time to 7 = all of the time). The ISE is scored by first summing the sub-scores. Then, the number of completed items is subtracted, and this result is multiplied by 100. Possible scores range from 0 to 100 with higher scores indicating a greater severity of problems. Scores below 30 indicate absence of a clinically significant problem, scores between 30 and 70 suggest the presence of a clinically significant problem, and scores above 70 indicate severe stress with an increased risk of considering or using some type of violence to deal with problems.

### Psychopathology

Psychopathology was rated by means of the Positive and Negative Syndrome Scale (PANSS) [42]. For statistical analysis, the PANSS was divided into four dimensions according to the Chen et al. model: positive, negative, affective, and cognitive [43].

### Cognition

#### Premorbid intelligence

Premorbid intelligence was assessed with the Mehrfachwahl-Wortschatz-Intelligenztest (MWT-B) [44], a multiple choice vocabulary test consisting of 37 lines, each comprising five words: one is a regular, lexically correct word from the dictionary, while four are fictitious. Study participants are asked to find the correct word and to underline it. The raw value of the result is made up of the number of correctly recognized words and is then compared to a representative sample. By evaluating the overall table score, the percentile rank and the intelligence quotient can be determined.

#### Verbal learning and memory

Verbal learning and memory were assessed using the Münchner Gedächtnistest (MGT), a German version of the California Verbal Learning Test [45]. The test consists of 2 word lists (each with 22 terms), that can be assigned to 4 conceptual categories: beverages, clothing, fruits, and tolls. The first list is read five times and study participants are asked to reproduce as many terms as possible after each presentation (verbal learning). Then, the second list is presented once. This presentation is followed by free and cued recall trials of the first list. At the end of the test, another word list, consisting of the items in the first list and various distraction items, is presented. Participants must decide whether the items were included in the first list (recognition). The variables of interest were the total number of words recalled in the most productive of the five learning trials (MGT max), acquisition (= difference between MGT max and the total number of words recalled in the first trial), retention (percentage of words recalled in the free-recall condition after a delay of 20 min based on the total number of words recalled in the most productive of the five learning trials), and recognition.

#### Visual memory

Visual memory was assessed with the Benton Visual Retention Test (Form F, M administration) (BVRT) [46]. First, 15 ink figures composed of geometric shapes are presented for 10 s. Subsequently, each of these figures has to be identified among three others. The number of correctly recognized items is calculated, the maximum score is 15.

#### Executive functioning

Verbal fluency was assessed with the Regensburger Wortflüssigkeits-Test (RWT) [47]. Study participants were asked to generate as many words as possible within the category of animals and as many words as possible starting with “S” in each of two 60-s trials. The number of correct words generated in each category was measured and compared with a norm table.

In addition, executive functioning (cognitive flexibility and problem-solving skills) was assessed using the 64-card computerized Wisconsin Card Sorting Test (WCST) [48]. Four cards with geometric symbols that differ in shape, color, and number are presented and study participants have to assign another card to one of the four target cards. They are not informed about the criterion for assignment (shape, color, number) and are requested to sort each of the cards. Feedback on the correctness of each assignment is given. Ten correct responses are required before the category is shifted to the next one. Continued assignment to a category after the matching principle has been switched is classified as a perseverative error. The variables of interest were the number of categories achieved and the percentage of perseverative errors.

#### Alertness

Alertness was assessed using subtest 1 out of the Test for Attention Performance (TAP) [49], which measures reaction times with or without an acoustic signal (“tonic alertness”). A cross appears in the middle of the screen and study participants are requested to press a button as rapidly as possible. The interval between warning and imperative stimuli varies randomly between 300 and 700 ms. The test is carried out according to an ABBA design (A: without acoustic signal, B: with acoustic signal) with 20 stimuli per condition. Next to the median reaction times in both conditions, we calculated their difference divided by the median reaction time of the total test (“phasic alertness” = ability to enhance the level of attention in expectation of a high-priority stimulus).

#### Working memory

Working memory was assessed using subtest 2 out of the TAP. 100 mixed single-digit numbers are presented consecutively on the screen. A number matching the penultimate one requires to press a button as quickly as possible. Median reaction time, the number of correct answers, and the number of errors were calculated.

### Statistical methods

The main purpose of the statistical analysis was an investigation of the effects of psychopathology (PANSS total score and components) and cognition (tests described above) on self-esteem (ISE). In the first part of the analysis, correlation analyses were performed. As the majority of the variables involved showed significant departures from normality, Spearman rank correlation coefficients were used. The combined effects of psychopathology and cognition on self-esteem were analyzed by multiple linear regression analysis with the ISE score as the dependent variable. To adjust for potential confounding effects of important patient characteristics (age, sex, education, and duration of illness), these variables were entered into the model first, irrespective of statistical significance. In a second step, PANSS components and cognitive variables were entered using stepwise variable selection. To limit the number of independent variables, only those variables were considered which had attained a *p*-value ≤ 0.1 in the correlation analysis. *R*^2^ was used as a summary measure to describe the proportion of variance accounted for by the regression model. All statistical tests were performed at a 0.05 level of significance.

### Power analysis

The sample size of 70 patients is sufficient to detect, under standard conditions regarding type-one error (two-tailed *α* = 0.05) and power (1 − *β* = 0.8), Spearman rank correlations of *r* = 0.328 or greater. This is a medium effect size according to Cohen’s classification [50]. Under the same conditions as above, the sample size of 70 allows detection of an effect size of *f*^2^ = 0.116 in a linear regression analysis with up to 7 independent variables, when testing for the effect of one additional predictor. This is again a medium effect size.

## Results

### Patient characteristics

Demographic and clinical characteristics are summarized in Table 1. 70 outpatients (58.6% male) with a mean age of 43.3 years and a mean duration of illness of 14.4 years participated in the study. The majority were living independently (i.e. alone, with own family, or in a small group home), were single, and unemployed. Residual symptomatology was generally mild. In terms of the Chen et al. model of the PANSS, negative symptoms showed the highest mean score followed by affective symptoms. Most patients received monotherapy with a new-generation antipsychotic.

**Table 1:** Patient characteristics (*n* = 70)

Age, mean ± SD, years	43.3 ± 8.9
Sex, %, M/F	58.6/41.4
Education, mean ± SD, years	11.7 ± 3.8
Duration of illness, mean ± SD, years	14.4 ± 8.3
GAF score, mean ± SD	61.3 ± 17.7
PANSS score, mean ± SD	
Total score (range: 30–120)	54.7 ± 18.8
Components^a^ (range: 1–7)	Positive: 1.70 ± 0.86
	Negative: 2.16 ± 1.08
	Affective: 1.94 ± 0.76
	Cognitive: 1.60 ± 0.63
Antipsychotic treatment^b^ *N* (%)	FGA monotherapy: 4 (5.7)
	NGA monotherapy: 59 (84.3)
	FGA + NGA combined treatment: 2 (2.9)
	NGA + NGA combined treatment: 5 (7.1)
Partnership status, *N* (%)	Single: 53 (75.7)
	Married/stable partnership: 6 (8.6)
	Divorced/widowed: 11 (15.7)
Housing, *N* (%)	With original family: 12 (17.1)
	With own family: 11 (15.7)
	Alone: 36 (51.4)
	In a small group home: 11 (15.7)
Employment status, *N* (%)	Full time employment: 3 (4.3)
	Part time employment: 3 (4.3)
	Supported employment: 10 (14.3)
	Unemployed: 54 (77.1)

*GAF* Global Assessment of Functioning Scale, *PANSS* Positive and Negative Syndrome Scale

^a^PANSS components as defined by the four-factor model by Chen et al. [40]

^*b*^*FGA* first-generation antipsychotic, *NGA* new-generation antipsychotic

### Self-esteem

As shown in Table 2, about two-thirds of the study participants presented with a clinically significant problem with self-esteem. One patient achieved an ISE total score > 70 thereby indicating severe stress, whereas the remaining sample achieved unremarkable scores.

**Table 2:** Self-esteem (*N* = 70)

	Mean or *N*	SD or %	Min—max
ISE total score (0–100)^a^	34.7	16.0	8–77
ISE total score < 30 (normal)	25	35.7%	
ISE total score 30–70 (clinically significant)	44	62.9%	
ISE total score > 70 (severe stress)	1	1.4%	

*ISE* Index of self-esteem

^a^Rescaled to a range from 0 (best self-esteem score) to 100 (poorest self-esteem score)

### Cognition

According to percentile ranking with reference to a norm sample, patients showed above-average premorbid intelligence (MWT-B) and achieved below-average test results in the domains of executive functioning (RWT verbal fluency, WCST perseverative errors), alertness (TAP Alertness with and without acoustic signal), and working memory (TAP Working memory-reaction time). They did not substantially differ from population norms with respect to phasic alertness (see Table 3).

**Table 3:** Cognitive variables (*N* = 70)

Cognitive variable	Mean	SD	Min–max
Premorbid intelligence (MWT-B, percentile)	63.4	27.1	9–99.8
MGT max (raw score)^a^	10.3	3.2	4–16
MGT acquisition (raw score)^b^	5.7	2.4	2 – 11
MTG retention (%)^c^	73.9	22.4	0–100
MGT recognition (raw score)^d^	14.5	1.8	8–16
BVRT (raw score)^e^	12.0	2.4	5–15
RWT verbal fluency animals (percentile)	20.7	22.6	0–88
RWT verbal fluency “s” (percentile)	26.3	26.8	0–100
WCST categories achieved (raw score)^f^	5.2	1.6	0–6
WCST perseverative errors (percentile)	30.1	28.9	1–99
TAP alertness			
Without signal, median (percentile)	15.2	20.4	0–84
With signal, median (percentile)	14.3	19.4	0–73
Phasic alertness (percentile)	49.4	32.8	1–99
TAP working memory			
Median reaction time, ms (percentile)	28.9	29.2	0–96
Correct answers (raw score)^g^	9.4	3.5	0–15
Errors (raw score)	5.3	4.9	0–15

*MWT-B* Mehrfachwahl-Wortschatz-Intelligenztest, *MGT* Münchner Gedächtnistest, *BVRT* Benton Visual Retention Test, *RWT* Regensburger Wortflüssigkeits-Test, *WCST* Wisconsin Card Sorting Test, *TAP* Test for Attentional Performance

^a^Total number of words recalled in the most productive of the five learning trials. Possible scores range from 0 to 16; higher scores indicate better performance

^b^Total number of words recalled in the most productive of the five learning trials minus the total number of words recalled in the first trial. Possible scores range from 0 to 16; higher scores indicate better performance

^c^Percentage of words recalled in the free-recall condition after a delay of 20 min based on the total number of words recalled in the most productive of the five learning trials. Possible scores range from 0 to 100; higher scores indicate better performance

^d^Possible scores range from 0 to 16; higher scores indicate better performance

^e^Possible scores range from 0 to 15; higher scores indicate better performance

^f^Possible scores range from 0 to 6; higher scores indicate better performance

^g^Possible scores range from 0 to 15; higher scores indicate better performance

### Association of self-esteem with sociodemographics, residual symptoms, and cognitive variables

There were no significant correlations between the sociodemographic variables assessed (age, sex, education, duration of illness, GAF score, partnership and employment status) and self-esteem (|*r*| < 0.15 and *p* > 0.1 for all variables).

Correlations of patients’ self-esteem with residual symptoms of the disorder and cognitive variables are presented in Table 4. Next to a weak but significant association with both the total score and the negative component of the PANSS, the ISE total score correlated moderately with affective symptoms (less self-esteem in study participants with more severe residual symptomatology). In addition, the ISE total score showed a correlation of moderate size with premorbid intelligence, verbal learning (MGT max), and working memory as well as a weak association with the acquisition of verbal material (MGT acquisition), visual memory (BVRT), and verbal fluency (RWT) (less self-esteem in study participants with better cognitive test performance).

**Table 4:** Association of self-esteem (ISE) with residual symptoms (PANSS) and cognitive variables, Spearman rank correlation (*n* = 70)

	Self-esteem
	ISE total score^a^
*PANSS scale/component*^b^	
Total score	0.290*
Positive	0.227
Negative	0.282*
Affective	0.519**
Cognitive	0.102
*Cognitive variable*	
Premorbid intelligence (MWT-B, percentile)	0.417**
MGT maximum (raw score)	0.368**
MGT acquisition (raw score)	0.276*
MTG retention (%)	0.153
MGT recognition (raw score)	0.106
BVRT (raw score)	0.263*
RWT verbal fluency animals (percentile)	0.124
RWT verbal fluency “s” (percentile)	0.252*
WCST categories achieved (raw score)	− 0.052
WCST perseverative errors (percentile)	− 0.171
TAP alertness	
Without signal, median (percentile)	0.075
With signal, median (percentile)	0.054
Phasic alertness (percentile)	− 0.012
TAP working memory	
Median reaction time, ms (percentile)	0.119
Correct answers (raw score)	− 0.029
Errors (raw score)	− 0.315**

*ISE* Index of self-esteem, *PANSS* Positive and Negative Syndrome Scale, *MWT-B* Mehrfachwahl-Wortschatz-Intelligenztest, *MGT* Münchner Gedächtnistest, *BVRT* Benton Visual Retention Test, RWT = Regensburger Wortflüssigkeits-Test, *WCST* Wisconsin Card Sorting Test, *TAP* Test for Attentional Performance

*p < 0.05 (2-tailed)

**p < 0.01 (2-tailed)

^a^Higher scores indicate lower levels of self-esteem

^b^PANSS components as defined by the four-factor model by Chen et al. [44]

### Results of multiple linear regression analysis

The combined effects of residual symptoms and cognitive variables on self-esteem were analyzed by multiple linear regression analysis, a summary of which is displayed in Table 5. After adjustment for age, education, and duration of illness, out of the potential predictors investigated female sex, affective symptoms, and premorbid intelligence showed a significant effect on patients’ self-esteem. The positive regression coefficients indicate that female sex as well as a higher degree of affective symptoms and premorbid intelligence were associated with poorer self-esteem.

**Table 5:** Effects of residual symptoms and cognitive variables on self-esteem (ISE total): findings of multiple linear regression analysis^a^

Variables	Beta	Standard error	Standardized beta	*t*	*p*-value
Patient characteristics, included to adjust for confounding					
Age	− 0.091	0.201	− 0.047	− 0.451	0.653
Education (years)	0.301	0.426	0.069	0.707	0.482
Duration of illness (years)	− 0.028	0.223	− 0.013	− 0.124	0.902
Significant predictor variables					
Sex (female vs male)	6.523	3.148	0.188	2.072	0.043
PANSS affective (Chen component)	12.828	1.914	0.604	6.701	< 0.001
Premorbid intelligence (MWT-B, percentile)	0.250	0.057	0.412	4.408	< 0.001
Variables not included in the model (not significant)					
PANSS negative (Chen component)	–^b^	–^b^	− 0.019	− 0.177	0.860
MGT max (raw score)	–^b^	–^b^	0.124	1.193	0.238
MGT acquisition (raw score)	–^b^	–^b^	0.152	1.588	0.118
BVRT (raw score)	–^b^	–^b^	0.052	0.415	0.680
RWT verbal fluency “s” (percentile)	–^b^	–^b^	0.124	1.171	0.247
TAP working memory errors (raw score)	–^b^	–^b^	0.058	0.615	0.541

*ISE* Index of Self-Esteem (higher scores indicate lower levels of self-esteem), *PANSS* Positive and Negative Syndrome Scale, *MWT-B* Mehrfachwahl-Wortschatz-Intelligenztest, *MGT* Münchner Gedächtnistest, *BVRT* Benton Visual Retention Test, *RWT* Regensburger Wortflüssigkeits-Test, *WCST* Wisconsin Card Sorting Test, *TAP* Test for Attentional Performance

^a^Model information: *R*^2^ adjusted = 0.491, *F* = 10.8, *df* = 6, *p* < 0.001

^b^Only standardized beta coefficients of these variables are displayed, as unstandardized betas of non-significant variables are not shown in the SPSS output

## Discussion

The present study was conducted to investigate the relationship between self-esteem and residual symptoms/neurocognitive functioning in clinically stable outpatients with chronic schizophrenia. Next to affective symptoms and premorbid intelligence, female sex emerged as a significant predictor of lower self-esteem, which corroborates the findings of earlier studies both in individuals with schizophrenia [7, 26, 28] and healthy subjects [51, 52].

As PANSS scores and sociodemographic factors show, this was a sample with some residual symptoms living in a stable social environment. We therefore were able to study the persistent impairments associated with this disorder, rather than the transient changes related to episodes of psychotic relapse. However, selecting a sample in this way clearly limits the generalizability of the data, since those who stop attending outpatient services are not included.

Next to a generally lowered self-esteem in people suffering from mental health disorders (MHD) compared to the general population, Silverstone and Salsali [53] found intermediate levels of self-esteem in individuals suffering from psychotic disorders compared to those with other psychiatric conditions. Accordingly, our finding of a mean ISE score of 34.7, i.e. a score in the lower range of values indicating a clinically significant problem with self-esteem, is not surprising and indicates that the sample included in this study might be comparable with outpatients treated in other countries. However, the fact that merely one-third of the study participants achieved ISE scores below 30 and thereby demonstrated good self-esteem emphasizes the relevance of strengthening this psychological characteristic in individuals suffering from schizophrenia spectrum disorders, even in times of relative absence of symptoms.

Previous studies have consistently shown that the performance of people with schizophrenia on important components of cognition is up to two standard deviations below the mean of healthy control subjects and is strongly associated with impairments in functioning [54, 55]. Our study sample’s performance on a comprehensive neuropsychological test battery clearly corroborates these earlier findings. Similarly, the association between patients’ self-esteem and residual symptoms of the disorder is in line with the results of other studies [8, 11, 12, 38]. Of note, the strongest correlation was detected with the affective component of the PANSS, which fits with a recent report by Demirkol et al. [56] who had used a specific instrument to measure the level and severity of comorbid depression in schizophrenia, the Calgary Depression Scale for Schizophrenia [57]. As mentioned above, self-disturbances as experienced by patients themselves may also be of particular relevance in this context [18–21]. Importantly, Bleuler already considered affect to be one of the four core characteristics of schizophrenia [58] and a recent study found significant depression in around one-fifth of patients during the phase of clinical remission, again with an adverse impact on various outcome domains [59]. Accordingly, our finding of residual affective symptoms predicting self-esteem is consistent with previous research and emphasizes the necessity to assess and manage depression during all phases of schizophrenia to support outcomes and to prevent further consequences [60].

Study participants’ self-esteem was negatively associated with premorbid intelligence as well as with performance in the domains of verbal learning and memory, visual memory, working memory, and verbal fluency. At first sight, this seems remarkable, since one would expect people with more pronounced neurocognitive impairment to have relatively lower self-esteem. However, as hypothesized by Wittorf et al. [30], a self-serving bias might protect people with distinct cognitive deficits from threats to their self-esteem and similarly, lack of insight, not measured in this study, may also have contributed to this finding. Following these considerations, we hypothesize that people with higher premorbid intelligence and less cognitive deficits may be more aware of their limitations caused by the illness, which, in turn, may result in a reduction in self-esteem. The sample investigated in this study consisted of stabilized individuals with chronic schizophrenia with relatively low PANSS scores. Thus, our findings corroborate previous research, according to which good symptomatic outcome is not necessarily associated with recovery [61, 62].

The current study also has some limitations. First, we used a cross-sectional design and included a sample of clinically stable patients to investigate a number of dynamic issues. Longitudinal designs clearly allow a more rigorous causal assessment of whether residual symptoms of the disorder and neurocognitive deficits influence self-esteem and to determine how the associations of these factors interact and change over time. Second, the MWT-B, which has been used to assess premorbid intelligence, is exclusively based on measures of verbal performance, which could be a bias for individuals who perform better in reasoning and spatial tasks. Third, there are a variety of other issues not considered in the current study and likely to have an influence on self-esteem, e.g., socio-cognitive functioning or family attitudes. Similarly, we could not investigate the impact of the type of antipsychotic medication on study results since most participants were on monotherapy with a new-generation antipsychotic drug. Lastly, self-esteem was assessed using a self-report questionnaire, which may be inferior to interview-based methods [63]. Notwithstanding these limitations, the present data provide evidence that a large proportion of individuals with chronic schizophrenia present with low self-esteem even when clinically stable. Our findings therefore reemphasize the relevance of promoting self-esteem as part of a comprehensive treatment of schizophrenia. In this regard, interventions such as solution focused group psychoeducation [64], metacognitive training [65], or cognitive behavioral therapy [66] have been shown to have a positive effect. In addition, our findings implicate that therapeutic strategies that specifically target affective symptoms of schizophrenia may have a beneficial impact on patients’ self-esteem.

## Data Availability

The anonymized dataset is available from the corresponding author on reasonable request.
